# Human Herpesvirus 6 (HHV-6)-Associated Longitudinally Extensive Transverse Myelitis Preceded by Mycoplasma pneumoniae Infection in an Immunocompetent Adult: A Report of a Unique Case

**DOI:** 10.7759/cureus.101124

**Published:** 2026-01-08

**Authors:** Bassem Al Hariri, Ahmad E Alharafsheh, Mohamed R Aboukamar, Joudi Alhariri, Abdulqadir J Nashwan

**Affiliations:** 1 College of Medicine, Qatar University, Doha, QAT; 2 College of Medicine, Weill Cornell Medicine - Qatar, Doha, QAT; 3 Department of Internal Medicine, Hamad Medical Corporation, Doha, QAT; 4 Department of Pharmacy, Hamad Medical Corporation, Doha, QAT; 5 Department of Infectious Diseases, Hamad Medical Corporation, Doha, QAT; 6 Department of Medical Education, Hamad Medical Corporation, Doha, QAT; 7 Department of Nursing and Midwifery Research, Hamad Medical Corporation, Doha, QAT

**Keywords:** ganciclovir, hhv-6, human herpesvirus 6, immunocompetent host, letm, longitudinally extensive transverse myelitis, meningoencephalitis, mycoplasma pneumoniae, transverse myelitis

## Abstract

In immunocompetent individuals, human herpesvirus 6 (HHV-6) is an uncommon cause of severe neurological illness. We describe the case of a 38-year-old man who had previously been in good health. He initially presented with fever and cough, followed by abrupt flaccid paraparesis, a T10 sensory level, and urinary retention. Neurological examination showed extensor plantar responses, hyperreflexia, and bilateral lower limb weakness. Magnetic resonance imaging of the spine demonstrated longitudinally extensive transverse myelitis (LETM) from T1 to T10. *Mycoplasma pneumoniae* was detected on initial serologic testing. The diagnosis of HHV-6 meningomyelitis was confirmed by lumbar puncture, which showed lymphocytic-predominant pleocytosis and a positive HHV-6 PCR result in the CSF. High-dose intravenous corticosteroids, azithromycin, and intravenous ganciclovir were administered, leading to successful treatment with notable neurological improvement and the ability to walk with assistance at discharge. This case highlights the diagnostic challenges of HHV-6, an emerging pathogen that can cause serious CNS complications such as LETM in immunocompetent hosts. It also raises the possibility of a combined immunomodulatory and antiviral therapeutic approach, even in the presence of a concurrent infection.

## Introduction

The beta-herpesvirus human herpesvirus 6 (HHV-6) is almost universally acquired by the age of two. Over 90% of children experience a primary infection, which usually presents as roseola infantum [1]. Following primary infection, the virus establishes lifelong latency in monocytes and macrophages, with sporadic reactivation, particularly in immunocompromised individuals. Encephalitis, myelitis, and other serious sequelae are well-documented consequences of HHV-6 reactivation in this population [2].

In contrast, severe neurological disorders caused by HHV-6 in immunocompetent individuals are extremely uncommon and poorly understood. Reported manifestations include meningitis, encephalitis, and acute disseminated encephalomyelitis [3,4]. Transverse myelitis (TM), particularly the longitudinally extensive form (LETM) affecting three or more contiguous spinal segments, is even rarer [5]. Diagnosis is challenging, often one of exclusion, and requires a high index of suspicion, with confirmation by PCR detection of HHV-6 DNA in CSF.

The central clinical question addressed by this case is whether acute LETM in an immunocompetent host represents a primary HHV-6 infection, reactivation triggered by a concurrent *Mycoplasma pneumoniae *infection via an immune-mediated (para-infectious) mechanism, or a synergistic interaction between the two pathogens. We present a rare and instructive case of an immunocompetent adult who developed acute LETM and meningoencephalitis in the context of co-detection of HHV-6 and *M. pneumoniae*. This case underscores the diagnostic complexity and supports consideration of a combined immunomodulatory and antiviral therapeutic strategy.

## Case presentation

A 38-year-old Pakistani man presented to the emergency room with a three-day history of moderate fever and a severe cough. He had no significant prior medical or surgical history. On the day of admission, he developed acute lower limb weakness, resulting in frequent knee buckling, urinary retention, and numbness extending up to the umbilicus. He was an occasional smoker but had no history of trauma, diabetes, hypertension, or known allergies.

On physical examination, his vital signs were stable: temperature 37.8°C, blood pressure 148/89 mmHg, heart rate 80 bpm, respiratory rate 18 breaths per minute, and SpO₂ 97% on room air. Systemic evaluation of the cardiovascular, respiratory, and abdominal systems was unremarkable. Neurological assessment revealed intact cranial nerves, and the patient was fully awake and oriented (GCS 15/15). Motor examination showed significant lower limb weakness (proximal 3/5, distal 4/5), with normal strength in the upper limbs. Deep tendon reflexes were brisk in all four limbs, accompanied by bilateral ankle clonus and extensor plantar responses (Babinski sign). Sensory testing confirmed a sensory level at T10 for light touch and pinprick. Initial laboratory investigations were notable and are summarized in Table 1.

**Table 1:** Initial laboratory investigations

Test	Result	Reference range
White blood cell count	9.4 × 10³/µL	4.0-11.0 × 10³/µL
C-reactive protein	5.8 mg/L	<5.0 mg/L
Potassium	3.5 mmol/L	3.5-5.1 mmol/L
Troponin-T	5 ng/L	<14 ng/L
HbA1c	5.50%	<6.5%
HIV Ag/Ab combo	Nonreactive	Nonreactive
Hepatitis B surface antigen	Nonreactive	Nonreactive
Hepatitis C antibody	Nonreactive	Nonreactive

The patient was placed under droplet precautions and started on empirical ceftriaxone. Azithromycin (500 mg orally once daily) was added after a nasopharyngeal swab tested positive for *M. pneumoniae *by PCR. The chest X-ray was unremarkable.

**Figure 1:**
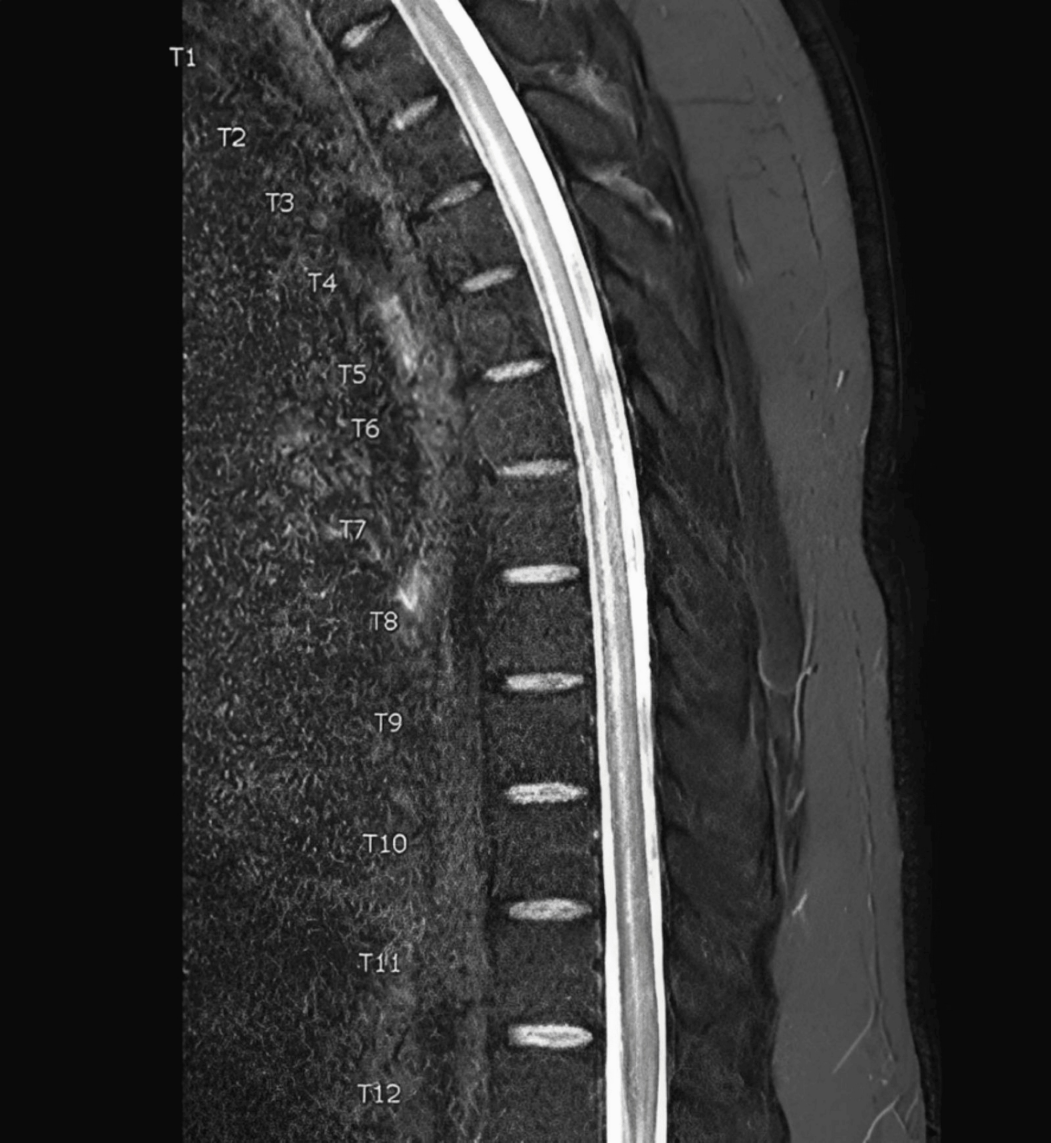
MRI of the thoracic spine Sagittal image demonstrating a long segment of hyperintensity within the spinal cord parenchyma, extending from T1 to T10 (arrows), consistent with LETM. LETM, longitudinally extensive transverse myelitis

An urgent MRI of the thoracic spine with contrast was performed, revealing a longitudinally extensive intramedullary T2/STIR hyperintensity extending from T1 to T10 vertebral levels, predominantly involving the anterior cord, consistent with LETM (Figure 1).

No cord compression or significant post-contrast enhancement of the cord was observed; however, patchy meningeal enhancement was noted in the upper cervical region. There was no diffusion restriction. A diagnostic lumbar puncture was subsequently performed (Table 2).

**Table 2:** CSF analysis HHV-6, human herpesvirus 6

Parameter	Result	Reference range (typical adult)	Interpretive note
Appearance	Clear, colorless	Clear, colorless	-
Total nucleated cells	525/µL	0-5/µL	Marked lymphocytic predominant pleocytosis, indicative of active inflammation, often viral or autoimmune
Lymphocytes	90%	40-80% (of total)	-
Neutrophils	4%	0-20% (of total)	-
Red blood cells	104/µL	0/µL	Suggests traumatic tap
Glucose	4.16 mmol/L	2.2-4.4 mmol/L	Normal
Protein	0.58 g/L	0.15-0.45 g/L	Elevated, supporting intrathecal inflammation
CSF culture	No growth	No growth	-
*Mycoplasma pneumoniae* PCR	Negative	Negative	Suggests respiratory, not CNS, infection
Herpes simplex virus 1/2 PCR	Negative	Negative	-
Varicella-zoster virus PCR	Negative	Negative	-
HHV-6 PCR	Positive	Negative	Confirms active HHV-6 infection within the CNS

A diagnosis of acute transverse myelitis, most likely para-infectious secondary to HHV-6, was made based on the clinical presentation, radiological features of LETM, and CSF findings. The patient received intravenous methylprednisolone (1 g daily) for five days. Following a positive CSF HHV-6 PCR, an Infectious Diseases consultation confirmed the viral etiology. Intravenous ganciclovir (5 mg/kg every 12 hours) was administered for a planned duration of two to three weeks, with the intent to transition to oral valganciclovir. The treatment duration followed established protocols for HHV-6 CNS disease in immunocompromised hosts and continued until significant clinical improvement was sustained, acknowledging the lack of standardized guidelines for immunocompetent patients.

The patient’s clinical course showed steady improvement. By the third day of steroid therapy, he was able to walk with assistance. At discharge, lower limb strength had improved to 4+/5, and voluntary urination was regained. He was discharged on a tapering regimen of oral prednisolone (starting at 60 mg daily) and oral valganciclovir (900 mg once daily). Serum testing for neuromyelitis optica (NMO-IgG) and myelin oligodendrocyte glycoprotein (MOG) antibodies was sent at discharge to exclude primary neuroinflammatory disorders; results were pending at the time of discharge and are discussed later.

## Discussion

This case illustrates two intersecting pathogenic processes: a pulmonary *M. pneumoniae *infection and a severe neurological illness caused by HHV-6. The temporal sequence suggests that the *Mycoplasma* infection may have acted as an immunological trigger, potentially disrupting immune homeostasis and allowing latent HHV-6 to reactivate and directly invade the CNS, resulting in meningoencephalitis and LETM. However, it is important to emphasize that this represents a correlation; a definitive causal relationship or proof of pathogenic synergy cannot be established from a single case report.

LETM is a severe neurological syndrome often associated with autoimmune conditions such as neuromyelitis optica spectrum disorder and MOG antibody-associated disease (MOGAD) [6]. Therefore, infectious causes must be thoroughly investigated, particularly in immunocompetent patients presenting acutely. HHV-6 is a well-recognized cause of limbic encephalitis in transplant recipients [2], but its role in causing LETM in immunocompetent individuals has been documented only in a few case reports [5,7]. Detection of HHV-6 DNA in the CSF, as observed in our patient, is considered indicative of active viral replication rather than latent infection [8].

The co-detection of *M. pneumoniae *adds complexity. *M. pneumoniae *is a common trigger for para-infectious neurological syndromes, including transverse myelitis, potentially through mechanisms such as molecular mimicry and autoimmune cross-reactivity [9]. In this case, the systemic inflammatory response induced by *Mycoplasma *may have impaired immune surveillance, facilitating HHV-6 reactivation and CNS invasion. A synergistic interaction between a bacterial infection and viral reactivation has been hypothesized previously [10].

The underlying pathological mechanism likely involved several interconnected processes. First, systemic inflammation from the respiratory *Mycoplasma* infection was present. Second, HHV-6 reactivation and neuroinvasion caused direct viral damage and localized inflammation within the spinal cord, as demonstrated by T2 hyperintensity from T1 to T10. Third, a concurrent meningeal inflammatory response was observed, indicated by cervical leptomeningeal enhancement on MRI. The CSF profile, lymphocytic pleocytosis with elevated protein, reflects this combined inflammatory state affecting both the spinal cord and meninges.

Treatment for HHV-6-related CNS disease in immunocompetent hosts is not standardized, as many cases are self-limiting. However, in severe presentations such as ours, antiviral therapy with ganciclovir or foscarnet is often used, extrapolating from evidence in immunocompromised populations [2]. Concurrent high-dose corticosteroid therapy targets the inflammatory component, which may be driven both by the viral infection and a para-infectious immune response. Our patient’s rapid and substantial clinical improvement with this combined regimen suggests a potential synergistic benefit, supporting its consideration in similar severe cases.

This case has several limitations. At the time of initial submission, serum NMO-IgG and MOG antibody results were pending. These results have since returned and were negative, strengthening the argument for HHV-6 as the primary cause in this immunocompetent patient, although other seronegative neuroinflammatory disorders cannot be entirely excluded. The possibility of HHV-6 chromosomal integration (ciHHV-6), which can lead to high viral DNA loads, was considered. However, the clinical context of acute myelitis with CSF pleocytosis favors active infection over chromosomal integration, which is often asymptomatic. Testing for ciHHV-6 via quantitative PCR on whole blood was not performed but may be considered in future cases. Long-term follow-up is essential to ensure full recovery and to monitor for relapses, which could indicate an underlying, initially undetected neuroinflammatory disorder.

## Conclusions

This report highlights that HHV-6 should be considered in the differential diagnosis of acute myelitis and meningoencephalitis in immunocompetent individuals, particularly when presenting as LETM. A comprehensive infectious workup, including CSF PCR for HHV-6, is essential for establishing an accurate diagnosis, especially in the presence of a concurrent infection. These conclusions are cautiously framed: while the clinical presentation is consistent with a para-infectious or immune-mediated process potentially triggered by *M. pneumoniae *and mediated by HHV-6, definitive causation cannot be established. The excellent clinical outcome in this case offers a practical model for managing similar severe neurological presentations using a combination of immunomodulatory and antiviral therapy. Nevertheless, to develop formal treatment guidelines for this rare but serious condition, additional case reports and systematic research are needed.
